# The Influence of System Dynamics Resource Sharing on Collaborative Manufacturing Efficiency—Based on the Multiagent System and System Dynamics Method

**DOI:** 10.3389/fpsyg.2022.837171

**Published:** 2022-05-09

**Authors:** Xiaoxia Zhu, Xu Guo, Hao Liu, Shuang Li, Xiaohong Zhang

**Affiliations:** ^1^Institute of Advanced Studies in Humanities and Social Sciences, Beijing Normal University, Zhuhai, China; ^2^School of Economics and Management, Yanshan University, Qinhuangdao, China

**Keywords:** Internet, collaborative manufacturing, resource sharing, system dynamics, multiagent

## Abstract

To improve the problems of inconvenient communication in the manufacturing industry, the ineffective use of resources, and the inability to efficiently complete manufacturing tasks, resource sharing has become an important model to promote the transformation and upgrading of the manufacturing industry. We used multiagent modeling to construct a resource-sharing model and take Baosteel as the micro background and the manufacturing industry as the macro background. Under this model, we discovered the effect of resource sharing on the efficiency of intelligent manufacturing under network collaboration through system dynamics research. We built and simulated a dynamic model of system dynamics that couples the two backgrounds and have given policy suggestions according to the simulation result.

## Introduction

### Manufacturing Operation Mode

As the Internet has rapidly developed, all types of traditional enterprises have combined, which brings infinite convenience to people’s lives. However, because of the characteristics of the manufacturing industry, the combination of the Internet and the manufacturing industry remains limited. The traditional manufacturing operation mode is to use enterprise resources and a few partners to outsource production activities. These basic activities cannot transcend geography and new enterprise thresholds. Accordingly, research on manufacturing resource management platforms came into being. Beginners used the Internet technology to build, but its disadvantages gradually appeared. Guo et al. (2000) introduced agents into manufacturing systems and used multiagent systems to construct a manufacturing resource collaborative management platform. Enterprises in different regions cannot cooperate well and find it difficult to accurately understand unfamiliar enterprises. Therefore, an important research issue has become how to transcend the boundaries of the manufacturing industry to share technologies, equipment, and services. Transcending these boundaries will allow “shared manufacturing” to optimally allocate and efficiently utilize manufacturing resources.

### Research Progress of System Dynamics

This article uses system dynamics to study the influencing factors of innovation in resource sharing. System dynamics was founded in 1956 by Professor Forrester of the Massachusetts Institute of Technology. Forrester (1958) proposed a system simulation method to analyze enterprise problems, such as production and inventory management. Since then, it has become a horizontal discipline that integrates the natural and social sciences and has been widely used in many fields. Naill (1992) and Naill et al. (1992) used system dynamics to analyze national energy policy planning and the cost of reducing energy policy. Domestic system dynamics research has been ongoing since the start of the 21st century. Wang (1984) led the development of system dynamics. He used books to introduce it to Chinese academic circles. In subsequent decades, Chinese scholarly achievements blossomed; these scholars applied system dynamics at both the macro and micro levels and to various industries. Regarding management, Ning and Liu (2004) studied industrial cluster evolution using system dynamics. Hu et al. (2006) used system dynamics to examine the problems of the enterprise life cycle. Cai et al. (2008) researched early warnings of enterprise financial crises based on system dynamics. Sun et al. (2008) employed system dynamics to explore the performance management of dynamically balanced scorecards in manufacturing enterprises. Lai et al. (2009) utilized system dynamics to solve logistical outsourcing problems and found that it was also very effective in measuring the innovation effect. Dong et al. (2009) studied the original innovation ability of enterprises based on system dynamics. Xu and Zhao (2011) examined the uncertainty of enterprise original innovation. Hu et al. (2011) investigated the influencing factors of industry–university–research cooperation under open innovation. Xu et al. (2012) explored the influencing factors of government research and development (R&D) subsidies and enterprise R&D behavior based on system dynamics. Lin and Guo (2017) carried out system dynamics-based simulation research on enterprise network public opinion propagation based on the characteristics of the communication subject. Zhao and Guo (2016) used system dynamics to control the logistics costs of enterprises. Because of its extraordinary adaptability, system dynamics have solved some innovation problems well. For example, Li and Qi (2017) considered the system dynamics of enterprise open innovation community management from the perspective of the innovation value chain. Gong and Li (2020) discussed the role of emotional communication and information exchange in the evolution of creative performance. Chen and Wang (2020) analyzed the impact of three systems on the innovation performance of high-tech enterprises from the perspective of knowledge spillover.

The existing research has not given enough attention to the manufacturing value network, innovation input factors, and macro-level government behavior factors at the macro- and micro-comprehensive levels. Based on the current research methods of scholars on innovation issues, this article expands the existing research on manufacturing synergy efficiency from two dimensions. First, we used systematic thinking to further study the innovation of manufacturing resource sharing at the macro and micro levels. Second, combining the internal coupling and external synergy effects of the manufacturing industry, we quantitatively analyzed the synergistic efficiency of manufacturing resource sharing.

To extend the above two dimensions, the first part of this article expounds on the research progress of the manufacturing production mode and system dynamics. The second part introduces multiagent and system dynamics methods and describes the construction process of the cloud service platform and system dynamics model. The third part outputs the manufacturing flow diagram under the micro and macro scenarios and quantitatively studies the influencing factors of collaborative manufacturing efficiency. The fourth part discusses the research methods, challenges, and limitations and proposes suggestions for improving the efficiency of collaborative manufacturing in the manufacturing industry. The fifth part contains a summary of the full-text research.

## Materials and Methods

### Methods

#### Multiagent Systems

An agent is a software or hardware entity capable of independent activities. It has some characteristics—such as autonomy, sociality, reactivity, initiative, mobility, honesty, rationality, and adaptability—and is widely used in various fields. Multiagent systems use multiple agents to complete a certain work, and each agent communicates with other agents and coordinates and shares all of the system tasks.

Complete internal enterprise processes, such as information registration and resource integration, are combined, as are complete macro manufacturing enterprise processes, such as communication and purchase transactions. Likewise, the multiagent system advantages are used. In these ways, the resources distributed in different regions, organizations, or systems can be organized to complete a specific task through networked manufacturing resource collaboration. The collaborative problem of the manufacturing business lies in spatiotemporal coordination. Different agents can solve this problem well.

#### System Dynamics

System dynamics is a discipline that analyzes and studies the information feedback system. It is also an interdisciplinary subject for understanding and solving system problems. Its purpose is to determine the influencing factors of the problem by analyzing the causal feedback relationship between the system elements to provide the basis for resolving the issue.

Using system dynamics to study problems is a process of decomposition and synthesis, repeated cycles, and the gradual realization of the research purpose. At the same time, system dynamics is based on a study of the whole and the relationship among the parts of the whole. As such, it replaces the traditional element view with the overall view and is suitable for multiple complex interactions that occur in the collaborative process between manufacturing enterprises. System dynamics are introduced into the research on collaborative manufacturing efficiency.

### Platform Construction

#### Research Framework

**FIGURE 1:**
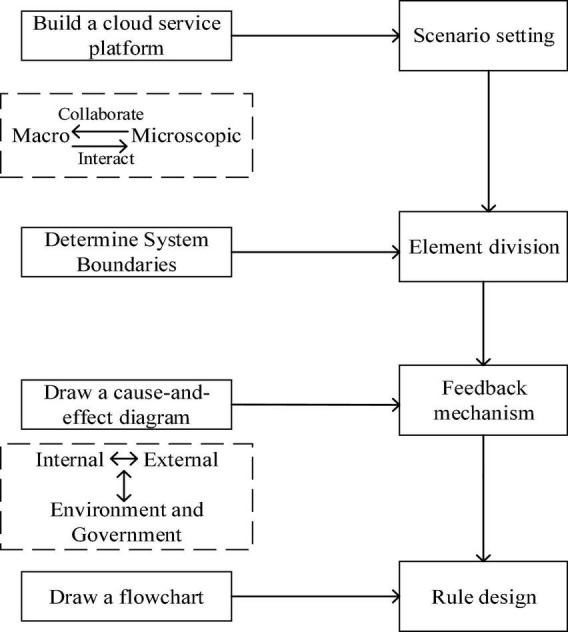
Third part of the research framework.

Scenario setting is performed through a cloud service platform to clearly understand the resource-sharing behaviors among manufacturing enterprises. After clarifying the resource-sharing process, we combined the principles of system dynamics to study the effect of resource sharing on the efficiency of collaborative manufacturing. Taking Baosteel enterprises as the research object and the collaborative transaction process of resource sharing as the basis, this study draws a coupling relationship between resource sharing and network collaborative manufacturing, analyzes the factors in the subsystem, and calculates the influencing effect between each factor. Then, we explored the internal coupling and external equilibrium effects of manufacturing resource sharing. The research framework is presented in Figure 1.

#### Enterprise Resource Sharing Based on the Multiagent System

**FIGURE 2:**
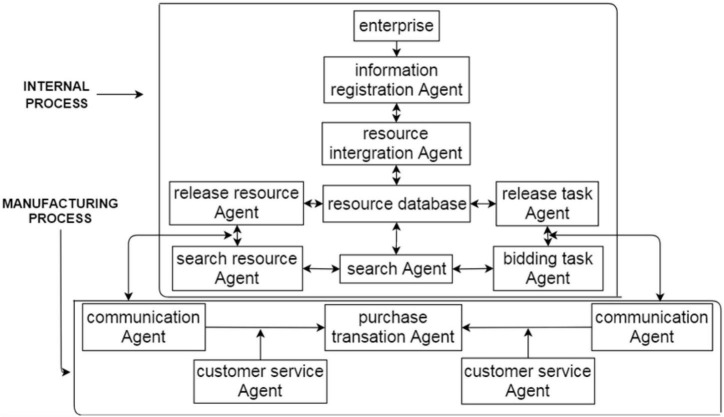
Cloud service platform.

The cloud service platform established by the multiagent system is shown in Figure 2.

The resource-sharing and cooperation relationship of manufacturing enterprises can be divided into internal and macro-manufacturing activities. In resource-sharing activities, each agent is assigned behavioral rules according to its essential attributes, and the agents act according to their own rules. Over time, the manufacturing system forms different scenarios.

(I) First, the micro-enterprise individual is the main body of resource sharing. The individual joins the multiagent system and develops resource-sharing behaviors with tens of thousands of enterprises. In this system, individuals carry out information registration, resource sharing, supply, and demand, and so on. These behaviors mainly need to be carried out by agents, such as information registration, resource integration, retrieval, release task, resource release, and bidding task agents. Among them, the information registration agent is the basis, and the resource integration and retrieval agents play intermediary roles by providing the information aggregated within the enterprise to the publishing resource and bidding task agents and cooperating with other manufacturing systems through communications between the publishing resource and the bidding task agents.
(1) Information registration agent: Enterprises that use this agent need to first register, and the information collection agent collects the enterprise’s information (enterprise name, address, resources, and business items).
(2) Resource integration agent: Enterprise-owned resources are entered into the resource database app. This agent is in charge of the classification and integration of resources in the resource library. The integration agent understands the nature and types of resources.
(3) Retrieval agent: The retrieval agent has two tasks. Enterprises can search for the required resources according to their own different needs, and enterprises can search or browse the tasks.
(4) Release task agent: Task enterprises can release some out-of-task packages.
(5) Release resource agent: Enterprises release their resources for other enterprises to seek cooperation.
(6) Bidding task agent: Enterprises look for tasks. When they find suitable and competent tasks, they express their desire to bid and do so along with their competitors. Then, they wait for the task enterprise to reply.

(II) The entire platform provides a public resource-sharing channel for the manufacturing industry. A single enterprise becomes a user in the platform, and each enterprise meets other enterprises’ resource or labor needs, which greatly increases resource-sharing efficiency within the manufacturing industry. From the perspective of the macro-manufacturing industry, transaction behavior occurs between individual users (enterprises), and the platform is managed through agents, such as communication, purchase transaction, and customer service maintenance agents. Among them, the communication agent purchases from the transaction agent, and the client service maintenance agent promotes the relationship between the two.
(1) Communication agent: When an enterprise selects a resource or task enterprise, the communication agent provides both enterprises with communication channels and opportunities for in-depth understanding.
(2) Purchase transaction agent: The cooperation between the two enterprises is enacted through the purchase transaction agent.
(3) Customer service maintenance agent: In the process of independent communication and transactions, some questions and even friction may arise. Customer service maintenance can help to solve the problems that arise on both sides.

Through the cloud service platform, we can clearly understand the resource-sharing behaviors among manufacturing enterprises. After clarifying the process, we used system dynamics to study the impact of resource sharing and other factors on the efficiency of collaborative manufacturing.

### System Dynamics Model

#### Method of Analysis

**FIGURE 3:**
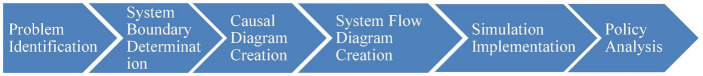
System dynamics flow.

The analysis process is illustrated in Figure 3.

After we chose the research methods of multiagent system and system dynamics, we further selected a suitable manufacturing enterprise to support the micro-level research. Baosteel Group has spent considerable resources promoting the construction of smart manufacturing. It has also established a resource-sharing management and control model that combines a professional focus and regional coordination. This model has become a new growth pole for the mutual support and coordinated development of manufacturing industries. To this end, we first studied the specific resource-sharing process, combined with the principle of system dynamics, by taking Baosteel enterprises as the research object based on the collaborative transaction process of resource sharing. Then, we drew a coupling relationship between resource sharing and network collaborative manufacturing. Then, we analyzed in detail the factors in the subsystem, calculated the influence effect among the factors, and explored the internal coupling effect of the manufacturing resource-sharing external equilibrium effect.

#### Problem Identification

(1) System boundary: The manufacturing system is divided into micro- and macro-manufacturing enterprises.
(2) A single enterprise internal organization structure subsystem: This includes the influence of resource sharing on the internal production and operation of an enterprise, the structure of production departments, and the organizational structure of the entire enterprise. The manufacturing enterprise relations subsystem includes the collaborative effect of resource sharing. The manufacturing eco-environmental subsystem includes the changes that resource sharing will bring to the whole manufacturing environment and operation, the impact on networked manufacturing resource sharing, and the impact of government macro-control on the manufacturing ecosystem.

#### Causal Diagram

System 1 includes internal enterprise behavior, such as the organizational behavior of learning ability training, material resource allocation, operation mode selection, and production mode selection. System 2 involves the manufacturing enterprise. System 3 comprises the manufacturing environment mode (Guo et al., 2019) and government behavior. The main causal relationships are illustrated as follows.

(1) Resource sharing → production flexibility → enterprise structure flattening → operating revenue → investment in scientific and technological innovation → network collaborative manufacturing efficiency.

Resource sharing is based on intelligent manufacturing, which has a definite impact on enterprise production processes. Virtual intelligence relates to the flexibility of production, and enterprises can adjust their organizational structures according to continuous market changes. Collaborative production reduces the management level and helps adjust the organizational structure of the enterprise by flattening the organizational structure. That is, it simplifies the organizational structure, reduces the management level, and establishes a compact and capable organizational structure. Collaborative production can enhance management efficiency, which increases operating income and promotes the efficiency of network collaborative manufacturing.

(2) Resource sharing → value network → number of enterprise cooperation units→ operating income → investment in scientific and technological innovation → network collaborative manufacturing efficiency.

Resource-sharing behavior encourages more transactional relationships so that the enterprise value network and enterprise cooperation increase while enterprise operating income improves. As income increases, more investments will be made in scientific and technological innovation, and efficiency will be promoted by network collaborative manufacturing.

(3) Resource sharing → network collaborative manufacturing efficiency → trust → resource sharing.

Resource sharing directly improves the efficiency of network collaborative manufacturing. This phenomenon promotes a virtuous circle that improves the trust of the entire manufacturing industry and results in a continual expansion of resource sharing.

(4) Resource sharing → production flexibility → enterprise structure flattening → operating revenue → manufacturing gross product → manufacturing operating environment → investment in scientific and technological innovation → network collaborative manufacturing efficiency.

Resource sharing promotes an increase in operating revenue; thus, it increases the gross product of the manufacturing industry, enhances the recognition of resource sharing in the whole manufacturing industry, increases investment in scientific and technological innovation, and improves the efficiency of network collaborative manufacturing.

(5) Resource sharing → organizational learning ability → enterprise structure flattening → operating income → investment in scientific and technological innovation → network collaborative manufacturing efficiency.

Resource sharing is equivalent to cooperation with more enterprises, and improving the learning ability of enterprises is conducive to improving network collaborative manufacturing efficiency.

(6) Resource sharing → production flexibility → production efficiency → operating revenue → investment in scientific and technological innovation → network collaborative manufacturing efficiency.

Resource sharing promotes production flexibility and improves production efficiency. Production efficiency increases operating income and improves efficiency.

**FIGURE 4:**
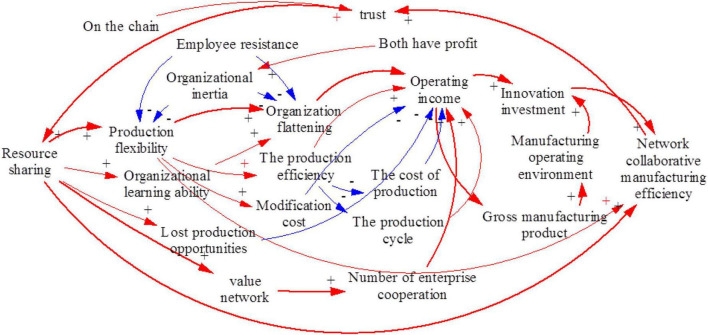
System causal diagram.

The system causal diagram is obtained, as shown in Figure 4.

Overall, enterprises share resources not to cooperate with familiar or long-term partners but to choose the enterprises with the most appropriate resources according to their specific business needs. Business diversification results in a broader circle of business contacts, promotes a production value network, and improves enterprise popularity in the manufacturing industry. It increases economic benefits, investment in collaborative manufacturing, and network collaborative manufacturing efficiency.

#### System Flow Diagram

The system causal diagram contains subsystems, including the single enterprise under the entire microscopic and macroscopic manufacturing subsystems and the subsystem under different manufacturing enterprises. Therefore, in the study of quantitative system flow diagrams according to the micro and macro levels, which are divided into internal flow diagrams and macro manufacturing, the influence of resource sharing on collaborative manufacturing efficiency is studied from two aspects.

##### Internal Flow Diagram of the Enterprise in the Microscopic Situation

Resource sharing within the enterprise mainly influences the efficiency of the production department, which affects the production department structure and makes the whole enterprise organizational structure flexible to improve collaborative manufacturing efficiency. The simulation undertaken in this article selects the Baosteel Group as an example. The Baosteel Group mainly engages in steel production, manufacturing, and sale, and it is among the world’s 500 compulsory molding enterprises with the typical operating characteristics of manufacturing enterprises.

## Descriptive Statistics

### Influencing Relationships

**FIGURE 5:**
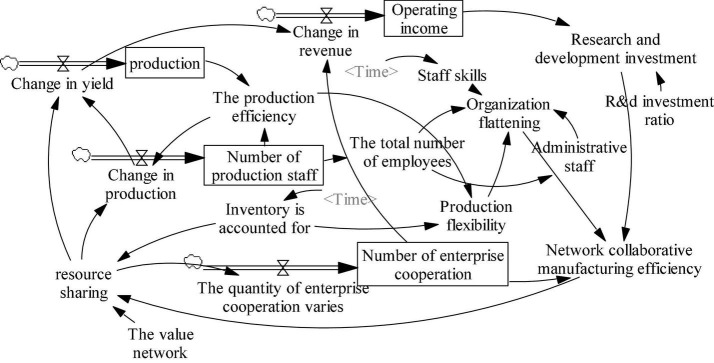
Internal enterprise flow diagram.

Based on the data in Table 1, the internal flow chart of the enterprise is obtained, as shown in Figure 5. The relationships between the variables and the main equation in the internal micro flow diagram of the enterprise are as follows.

(1) INITIAL TIME = 2008, FINAL TIME = 2019, STEP = 1 year.
(2) Value network = 2.5. The value network at the level of a single enterprise is expressed as the number of enterprise partners, which is set to 5 from the 2018 standard from the years 2008–2019, and the value network of the other years is obtained according to the standard.
(3) Flattening of the enterprise organizational structure = (total number of employees/number of administrative administrators) * 0.4 + staff skills * 0.3 + production flexibility * 0.3. The parameters are set according to the principle of importance sorting. (a) The reduction of management levels is the key factor that leads to enterprise flattening and the reduction in administrative personnel. Therefore, the weight of this part is greater than that of the other two parts. Based on experience, the weight is set to 0.4. (b) Enterprise flattening is also affected by the increase in employees’ skills. The workers’ skills are set to 1, and the annual growth rate is 5%. Based on experience, the effective weight is set to 0.3. (c) Production flexibility affects flattening, and it has the same probability of affecting flattening as employee skills have. Let the weight of production flexibility be 0.3. When the degree of enterprise flattening is higher, this is more conducive to the network collaborative manufacturing efficiency of the enterprise.
(4) Production flexibility = (labor productivity/100) * 0.6 + inventory proportion * 0.4. To increase production efficiency and reduce costs and inventory, flexible production in this article is defined as more production that is lean and intelligent (Guo et al., 2001). Set the parameters according to the principle of importance sorting. The quantitative measurement of flexible production is primarily measured by the input of enterprise technicians and automation equipment. According to management knowledge, the labor productivity ratio is the most important measure of production flexibility, and its impact is higher than the impact of the inventory ratio. For calculation convenience, this article sets the weight of labor production efficiency as 60% and the weight of the inventory ratio as 40%.
(5) Output = INTEG (changes in output, 22,813,000). The number 22,813,000 is the output in 2008 and is set as the initial value of the whole model: changes in output = –2,216,480 + 1,161.912 * change of production staff + 3,16,244 * resource sharing. The output of more mature enterprises is generally determined by the quantity demanded; therefore, the quantity demanded is used in this article to substitute for the output.
(6) Labor productivity = output/number of production staff.
(7) Number of production staff = INTEG (change of production staff, 26,327), change of production staff = 13,262.7 + 1,326.69 * resource sharing – 25.165 * production efficiency.
(8) Number of enterprises cooperating = INTEG (the change in the number of enterprises cooperating, 21). The initial value is the number of enterprises cooperating (21) in 2008. The change in the number of enterprise cooperation = –1.854482 + 0.369372 * resource sharing.
(9) Operating income = INTEG (change in operating income, 2003.32). The operating income in 2008 (2003.32) is the initial value of the entire model, and the change in operating income = 25.21294–1.820862 * number of enterprise cooperation + 5.43E – 05 * change in output.
(10) Enterprises’ investment in resource sharing = operating income * R&D investment ratio. R&D investment ratio = 2%. Given the average R&D expenses in the years 2008–2019, the R&D investment ratio was 2.04%. In addition, due to the different annual operating incomes and an upward trend, the annual investment ratio changed little, but from the overall 12-year trend, the R&D investment ratio increased to a certain extent; thus, the R&D investment ratio was 2%.
(11) Resource sharing = (1.209575 + 0.213763 * inventory ratio + 2.999953 * network collaborative efficiency) * value network. Resource sharing indicates the effective flow of resources between enterprises. In addition, network collaboration efficiency influences resource sharing; an increase in the value network greatly promotes resource sharing.
(12) Network collaborative efficiency = 0.329512-0.046255 * enterprise organizational flattening + 0.000509 * enterprise investment in resource sharing + 0.003445 * enterprise cooperation. Collaborative manufacturing efficiency is mainly manifested in enterprise profitability, and enterprises can obtain greater output levels with the same assets. In this article, the weighted average return on equity represents the collaborative manufacturing efficiency of networks.

**TABLE 1:** Partial data for the Baosteel Group from 2008 to 2019.

Year	2008	2009	2010	2011	2012	2013	2014	2015	2016	2017	2018	2019
Value network	2.5	2.6	2.5	3.1	3.2	3.1	3.0	3.0	3.8	4.2	5.0	5.2
Resource sharing	6.91	5.89	7.44	9.17	9.04	9.46	9.25	9.08	11.19	15.26	17.72	17.52
Production flexibility	7.45	7.22	7.70	8.59	9.62	8.95	8.81	9.01	9.95	11.03	11.26	12.20
Flatness degree	7.42	7.37	7.52	7.81	8.13	7.95	7.93	8.01	8.31	8.66	8.75	9.05
Production [ton]	22813000	20629080	23308511	25800000	23566000	21993100	21817000	22148300	24090000	46170000	47100000	47185000
Production personnel	26327	25468	25804	25839	20536	21694	22558	22745	21807	36734	37191	33652
Production efficiency [ton/person]	867	810	903	998	1148	1014	967	974	1105	1257	1266	1393
Number of cooperation between enterprises	21	22	21	26	27	26	25	25	32	35	42	44
R&D investment [100 million]	23.03	25.96	42.45	51.18	38.23	34.14	39.36	34.56	37.09	53.48	70.10	88.64
Proportion of R&D investment [%]	1.15	1.75	2.10	2.30	2.00	1.80	2.10	2.11	2.00	1.85	2.30	3.04
Operating revenue [100 million]	2003.32	1483.26	2021.49	2225.05	1911.36	1896.88	1874.14	1637.90	1854.59	2890.93	3047.79	2920.90
Network collaborative manufacturing efficiency [%]	6.99	6.27	12.95	7.02	9.52	5.29	5.16	0.90	7.68	12.24	12.70	7.05

*Source: Original data are from the 2009 to 2019 annual report of the Baosteel Group.*

#### Flow Chart of the Manufacturing Industry Under the Macro Situation

From the perspective of the system flow diagram for a single enterprise, the internal changes of the enterprise can be well observed. From the perspective of an outstanding single enterprise, it is equally important for the manufacturing industry and the government to understand the operational environment and affect the entire manufacturing ecosystem. In the macro-environment, the entire manufacturing industry and the government’s macro-control are essential because they mainly study the manufacturing industry and the government’s adjustment and change of some influencing factors.

**FIGURE 6:**
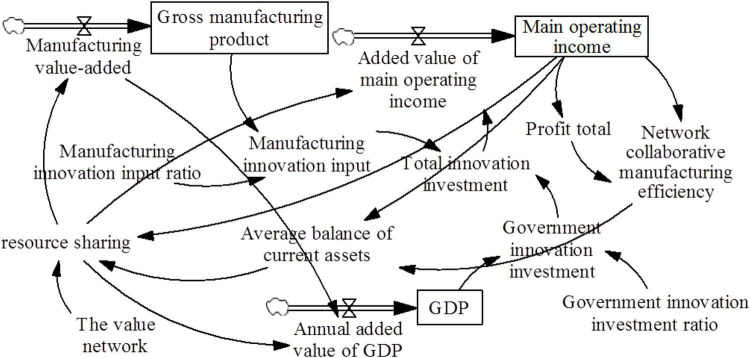
Macro flow diagram of the manufacturing industry.

Based on the data in Table 2, the macro flow chart of the manufacturing industry was obtained, as shown in Figure 6. The main factor relations and the main equation in the macro manufacturing flow diagram are as follows.

(1) INITIAL TIME = 2008, FINAL TIME = 2019, STEP = 1 year.
(2) Value network = 3.6. On the macro level, the value network is given in the number of enterprises above the manufacturing scale. The number of enterprises in 2010 compared with the highest number of enterprises in each year from 2008 to 2019 is regarded as the standard value of 5. The value network of this system takes the lowest value from 2008 to 2019.
(3) Manufacturing gross product = INTEG (manufacturing added value, 1,02,539.5), which indicates that the initial value of manufacturing gross product is 10,253.95 billion, and the manufacturing gross product = Σannual added value of sigma, manufacturing annual added value = 11163.9 + 453.0325 * resource sharing.
(4) GDP = INTEG (annual added value of GDP, 319245), which indicates that the initial value of GDP is 3,1924.5 billion, and GDP = Σannual added value of sigma GDP, annual added value of GDP = 1,34,142.7 + 1.133849 * added value of manufacturing industry – 10,426.41 * resource sharing.
(5) Main operating income = INTEG (added value of main operating income, 4,32,759.95), which means that the initial value of main operating income is 4,32,755.995 billion, and main operating income = added value of sigma main operating income, added value of main operating income = -421,152.3 + 0.770451 * total innovation investment + 52,836.33 * resource sharing.
(6) Investment in manufacturing innovation = gross product of manufacturing industry * manufacturing innovation input ratio. The innovation input ratio of the manufacturing industry was 4.56% on average each year from 2008 to 2019.
(7) Government investment in innovation = GDP * government investment in innovation ratio, and the government investment in innovation ratio was 0.41% on average from 2008 to 2019.
(8) Total profit = –738.7928 + 0.061555 * main operating income.
(9) Network collaborative manufacturing efficiency = total profit/(core operating income - total profit).
(10) Average balance of current assets = –9424.888–917037.3 * network collaborative manufacturing efficiency + 0.503167 * main operating income.
(11) Resource sharing = (current assets average balance) * value network. Resource sharing means that each enterprise can share. This article used the current asset turnover to quantify resource sharing. Liquid assets reflect the enterprise’s current asset turnover speed, which means that the enterprise has better liquidity intuitive performance.

**TABLE 2:** Important data for the above-scale manufacturing industry from 2008 to 2019.

Year	2008	2009	2010	2011	2012	2013	2014	2015	2016	2017	2018	2019
Manufacturing GDP [100 million]	102539.5	110118.5	130325.0	150597.2	161326.1	181867.8	195620.3	202420.1	214289.3	240505.4	264820.0	269000.0
Manufacturing R&D investment [100 million]	4165.2	5090.4	6260.1	5695.4	161326.1	181867.8	195620.3	202420.1	214289.3	240505.4	264820.0	13569.7
Manufacturing R&D investment ratio [%]	4.06	4.58	4.80	3.78	4.26	4.39	4.55	4.78	4.95	4.84	4.74	5.04
GDP [100 million]	319244.6	348517.7	412119.3	487940.2	538580.0	592963.2	641280.6	685992.9	740060.8	820754.3	900309.5	990865.1
Government R&D investment [100 million]	1088.9	1358.3	1696.3	1883.0	2221.4	2500.6	2636.1	3031.2	3140.8	3487.4	3978.6	4537.3
Average balance of current assets of enterprises above the scale [100 million]	165058.53	183687.26	220500.52	265861.02	304222.98	340777.26	376424.15	403471.30	429330.14	460106.71	483577.08	507578.3
Main operating income [100 million]	432759.95	471869.71	606330.07	729278.44	805662.29	901941.49	978229.96	992673.82	1047710.96	1019597.52	931189.9	943582.0
Value network	4.7	4.8	5.0	3.6	3.8	3.9	4.2	4.2	4.2	4.1	4.2	4.2
Resource sharing	9.3	9.1	9.8	9.9	9.5	9.5	9.3	8.6	8.7	7.8	6.9	6.6
Network collaborative efficiency	0.053	0.063	0.075	0.070	0.064	0.060	0.062	0.062	0.066	0.070	0.065	0.062

*Source: China Statistical Yearbook, number unit: 100 million.*

The above two structure flow diagrams were based on the system causal diagram, in which the set equation can determine the relationship between various variables. The data were obtained from the annual report of the Baosteel Group, the *China Statistical Yearbook*, and the calculations. Some constants were calculated based on known data, and some equation relations were calculated with the help of econometrics.

### Model Output and Response

The problem studied generates two flow diagrams due to dimensional differences, namely, one for macro manufacturing and the other for micro-enterprises. The contact points of the two flow diagrams are all enterprises, and the center is on the impact of resource sharing on network collaborative efficiency.

#### Flow Chart 1

The internal flow chart mainly studies the impact of resource sharing on various aspects of a single enterprise. Starting from resource sharing, it affects the number of cooperation enterprises, the number of production personnel, production flexibility, enterprise organizational flattening, output, operating revenue, R&D investment, and ultimately network collaboration efficiency. The two indicators of the final output and response of this flow chart are business income and network synergy efficiency, which directly or indirectly consider the benefits of resource sharing.

#### Flow Chart 2

The macro flow chart of the manufacturing industry studies the overall situation of many manufacturing enterprises, such as that depicted in Flowchart 1. It still affects the whole system from the perspective of resource sharing, affects the gross product and core operating income of the manufacturing industry, and thus affects network collaborative efficiency. The final output of this flow diagram demonstrates the corresponding network collaboration efficiency and operating revenue.

### Model Validation and Simulation

#### Effectiveness Test

(I) The original data used in the simulation were all from the 2008 to 2019 annual report of the Baosteel Group. The simulation parameters were configured according to relevant research and the actual situation. The authenticity of the data and the practicality of the parameters demonstrate the practical significance of the simulation results.
(II) In the adaptability test of the model, we know the adaptability of all aspects of the system model, which is in line with the policy and environment of all system aspects, and the policy analysis is of practical significance; therefore, the model has adaptability.
(III) The authenticity of the model is verified by comparing the actual data with the data generated by the system simulation. We thus observed the fitting degree of the enterprise’s operating revenue and network collaborative manufacturing efficiency. Table 3 shows that the output value of the simulation deviates to some extent from the real value, but the results reflect the yearly change in operating income and the influence of different factors on the network collaborative manufacturing efficiency, which has a reference value for policy analysis.

**TABLE 3:** Comparison of the real value and fitting values.

	Enterprise internal flow diagram				Macro manufacturing			
	Operating income		Collaborative efficiency		Operating income		Collaborative efficiency	
Year	Real value	Fitting values	Real value	Fitting values	Real value	Fitting values	Real value	Fitting values
2008	2003.32	2003.32	6.99%	7.94%	432759.95	432760	0.0530	0.0636
2009	1483.26	1999.00	6.27%	7.93%	471869.71	565175	0.0630	0.0641
2010	2021.49	2011.68	12.95%	7.91%	606330.07	646655	0.0750	0.0643
2011	2225.05	2034.78	7.02%	7.88%	729278.44	710145	0.0700	0.0644
2012	1911.36	2063.88	9.52%	7.84%	805662.29	763401	0.0640	0.0645
2013	1896.88	2096.07	5.29%	7.81%	901941.49	809876	0.0600	0.0646
2014	1874.14	2129.39	5.16%	7.77%	978229.96	851482	0.0620	0.0646
2015	1637.90	2162.46	0.90%	7.73%	992673.82	889415	0.0620	0.0647
2016	1854.59	2194.23	7.68%	7.69%	1047710.96	924483	0.0660	0.0647
2017	2890.93	2223.79	12.24%	7.65%	1019597.52	957262	0.0700	0.0647
2018	3047.79	2250.33	12.70%	7.61%	931189.90	988179	0.0650	0.0647
2019	2920.90	2272.99	7.05%	7.56%	943582.0	1017560	0.0620	0.0648

*As an iron and steel manufacturing enterprise, Baosteel Group is more vulnerable to the influence of uncontrollable factors, such as the overall economic development and industrial policies; thus, the actual situation and simulated situations will deviate to some extent.*

Table 3 shows a certain deviation between the fitted values and real values, but the results generally conform to the changing trend. Therefore, the flow diagrams of the two systems are feasible and can be simulated.

#### Simulation

In the two flowcharts, the effect and size of the output results are obtained by changing the value network and technological innovation input.

(I) Changing the value network (resource sharing) and the technological innovation input of enterprises changes the business income and efficiency of collaborative manufacturing in the enterprise network.
(1) Value network

**FIGURE 7:**
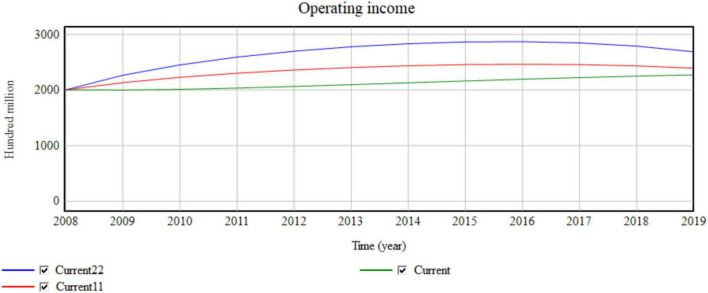
Influence of micro value networks on business income.

**FIGURE 8:**
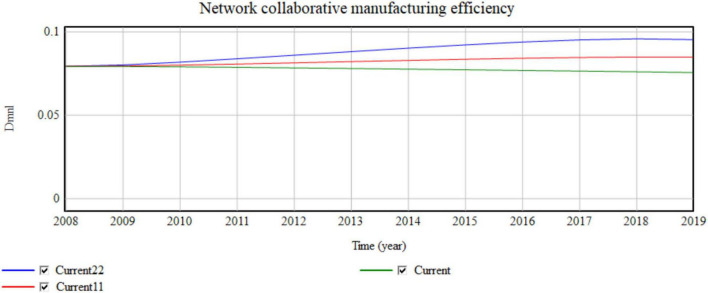
Influence of the micro value network on network collaboration efficiency.

In the microenvironment, the value network is divided into cooperation between enterprises. Businesses that work with more companies have greater trading opportunities and sharing conditions. The currency value network takes the lowest value of 2.5 of the value network from 2008 to 2019. Current11 takes 3 and Current22 takes 3.5 (Figures 7, 8).

Four cooperative enterprises are aggregated for each 0.5 value network added. The simulation results shown in Figures 7, 8 indicate that the yearly operating revenue increases by 134, 217, 268, …,100 million, and the yearly network collaborative efficiency increases by 0.0002791, 0.001007, and 0.0019599. Thus, the value network has an obvious impact on operating income.

The increase in partners provides more business opportunities, which greatly improves the business income of enterprises. In the conventional manufacturing environment, the limited cooperative operation objects of enterprises limit the maximum utilization of resources, which causes waste or shortage of resources. After resources are effectively shared, the business circle of a manufacturing enterprise greatly expands, which improves the efficiency of resource flow and reduces the excess or shortage of resources.

(2) Investment in technological innovation

**FIGURE 9:**
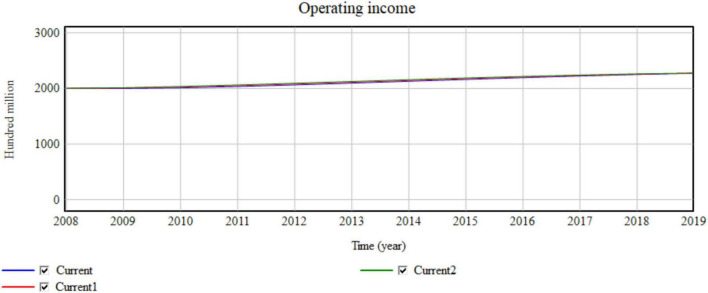
Influence of micro science and technology innovation investment on business income.

**FIGURE 10:**
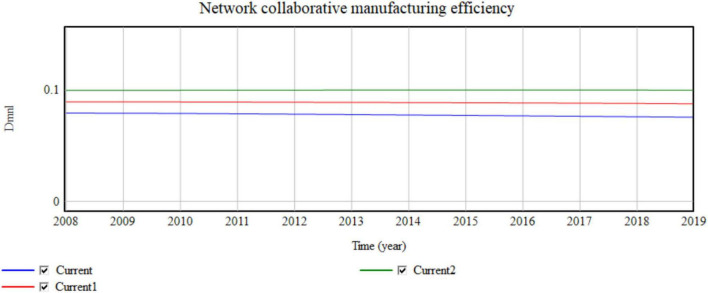
Impact of micro science and technology innovation investment on network collaboration efficiency.

Because of the enterprise nature of Baosteel Group, the current technology innovation investment ratio averages 2% from 2008 to 2019, Current1 increases by 3%, and Current2 increases by 4% (Figures 9, 10).

The simulation results shown in Figures 9, 10 indicate that for every 1% increase in the technological innovation of enterprises, the operating revenue increases by 7.72, 12.16, 14.44, …, 100 million. Thus, the network increases in collaborative efficiency by approximately 0.01 per year. Increasing the investment in science and technology innovation greatly influences network collaboration efficiency. Therefore, investment in scientific and technological R&D is extremely important for enterprise development, which includes not only technology improvement but also production equipment improvement.

(II) The investment in the value network (resource sharing) and scientific research and innovation in the macro-environment are changed, and the changes in the operating income of enterprises and the efficiency of enterprise network collaborative manufacturing are observed.
(1) Value network

**FIGURE 11:**
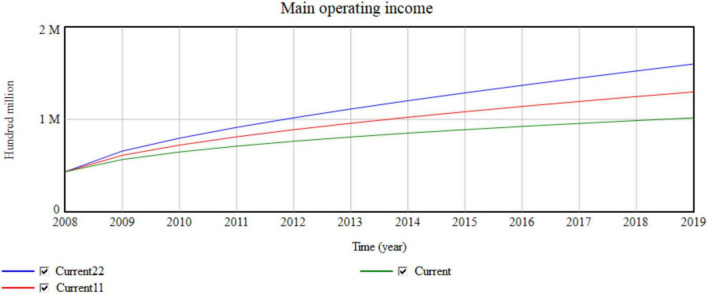
Impact of the macro value network on the main operating income.

The value network in the macro-environment represents the number of manufacturing enterprises. The value network increases from Current (3.6) to Current11 (3.9) and Current22 (4.2) (Figure 11).

Each 0.3-unit increase in the value network is equivalent to an increase of 26,000 enterprises. The simulation results shown in Table 4 and Figure 11 indicate that with the change in the value network, the collaborative manufacturing efficiency of the network increases by almost 0.0001. The increase in the main operating income increases with the yearly increase of 45,746, 749,63,…, 100 million. The increase in the number of enterprises improves the competitiveness of the entire manufacturing industry, which also improves the production efficiency and operating income of the industry. The increase in the efficiency of resource-sharing opportunities is also key to improving the efficiency of network collaborative manufacturing. The value network has a significant influence on revenue and synergetic efficiency in the macro-environment.

(3) Technological innovation investment in the manufacturing industry

**TABLE 4:** Influence of the macro value network on collaborative efficiency.

	2008	2009	2010	2011	2012	2013	2014	2015	2016	2017	2018	2019
Current	0.0637	0.0641	0.0643	0.0644	0.0645	0.0646	0.0646	0.0647	0.0647	0.0647	0.06547	0.0648
Current11	0.0637	0.0642	0.0644	0.0646	0.0646	0.0647	0.0648	0.0648	0.0649	0.0649	0.0649	0.0649
Current22	0.0637	0.0643	0.0645	0.0647	0.0648	0.0648	0.0649	0.0649	0.0650	0.0650	0.0650	0.0651

**FIGURE 12:**
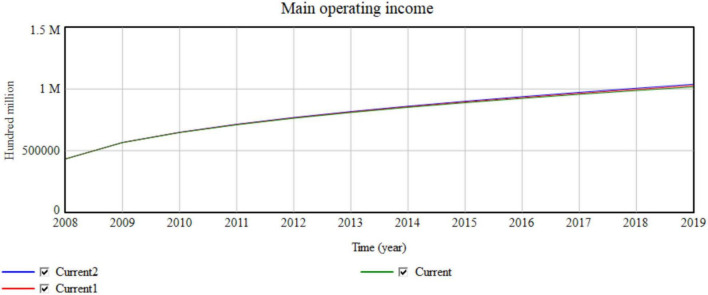
Impact of investment in scientific and technological innovation on the operating income of the macro manufacturing industry.

The technology innovation input ratio is set at an average of 4.56% of the actual technology innovation input ratio from 2008 to 2019. The technology innovation input ratio of System Current1 increases by 5.56% and that of Current2 increases by 6.56% (Figure 12).

The simulation results shown in Table 5 and Figure 12 indicate that for every 1% increase in the investment in scientific and technological innovation, the main operating income increases successively with the increase in annual income of 790, 1,488, …, 100 million. As the input value of scientific and technological innovation increases, the main operating income increases more obviously. Improving the science and technology investment also has a certain, non-obvious influence on the network collaborative manufacturing efficiency. For every 0.5% increase in the investment in science and technology innovation, the network collaborative efficiency increases by 0.00001. Thus, a large part of higher efficiency and better product quality depends on the improvement of technology, which ultimately increases the output value and improves efficiency.

(4) Government investment in scientific research and innovation

**TABLE 5:** Influence of innovation input on the network collaborative efficiency of the macro manufacturing industry.

	2008	2009	2010	2011	2012	2013	2014	2015	2016	2017	2018	2019
Current	0.0637	0.06411	0.06430	0.06441	0.06449	0.06456	0.06461	0.06465	0.06469	0.06472	0.06474	0.06477
Current1	0.0637	0.06411	0.06430	0.06442	0.06450	0.06456	0.06461	0.06466	0.06469	0.06472	0.06475	0.06478
Current2	0.0637	0.06411	0.06430	0.06442	0.06450	0.06457	0.06462	0.06466	0.06470	0.06473	0.06476	0.06479

**FIGURE 13:**
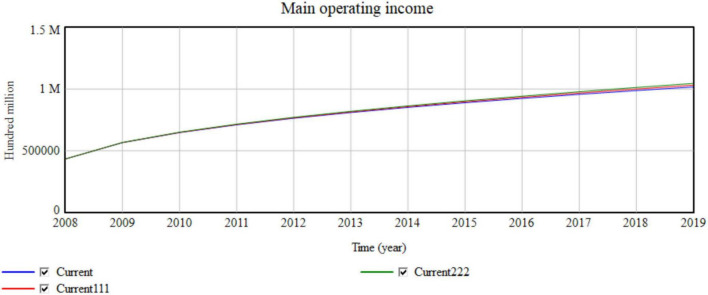
Impact of the macro government investment in science and technology innovation on operating revenue.

The government’s behavior is an important factor that affects the operation of the manufacturing industry. The government’s greatest support for enterprise resource sharing is to offer financial assistance for scientific and technological innovation. The initial government science and technology innovation investment ratio is 0.41%. Therefore, the current is 0.41%. Increasing the science and technology innovation investment results in a Current111 of 0.8% and a Current222 of 1.2% (Figure 13).

Table 6 and Figure 13 indicate that for every 0.4% change in the investment in science and technology, the network collaborative efficiency increases by 0.00001. For every 0.4% change in technology income, the main operating income increases with the increase of revenue in each year 959, 1,790, …, 100 million. Changing the government’s investment ratio for scientific and technological innovation amends the government’s support for manufacturing resource-sharing innovation.

**TABLE 6:** Influence of the macro government investment in science and technology innovation on network collaborative manufacturing efficiency.

	2008	2009	2010	2011	2012	2013	2014	2015	2016	2017	2018	2019
Current	0.06366	0.06411	0.06430	0.06441	0.06449	0.06456	0.06461	0.06465	0.06469	0.06472	0.06474	0.06477
Current 111	0.06366	0.06411	0.06430	0.06442	0.06450	0.06456	0.06462	0.06466	0.06469	0.06473	0.06475	0.06478
Current 222	0.06366	0.06412	0.06430	0.06442	0.06451	0.06457	0.06462	0.06467	0.06470	0.06474	0.06477	0.06479

The above simulation results yield the following conclusions.

(1) From the micro perspective, due to the objective finiteness, the change in variables caused by the change will be more obvious. The impact of the value network on operating income is greater than the impact of changing the investment in scientific and technological innovation. In addition, a single enterprise must increase its operating income so that it can increase revenue more quickly by cooperating with more enterprises to improve operating conditions. In addition, the impact of science and technology innovation investment on network collaborative efficiency is greater than the impact of the value network. The collaborative manufacturing efficiency can be improved. The priority is to upgrade the technology and equipment within the enterprise.
(2) From the macro perspective, due to the objectivity of larger sample formation, the change of the variable produces little change. According to the simulation results, changing the number of enterprises in the manufacturing industry has a significant impact on operating income and network collaborative manufacturing efficiency. In contrast, the influence of technological innovation of both enterprises and the government on business income and network collaborative manufacturing efficiency is relatively small. Therefore, the manufacturing industry must regulate and control the number of enterprises. Eliminating inferior enterprises can steadily expand the manufacturing market by ensuring quality to improve the possibility of resource sharing. However, increasing investment in scientific and technological innovation is also very significant for the upgrading of manufacturing industry efficiency. Although such an investment cannot greatly increase income, it will greatly promote the high-quality development of the manufacturing industry.

## Discussion

### Method Comparison

The comparison and analysis of the related references and the research in this article are depicted in Table 7.

**TABLE 7:** Comparative analysis of the research methods.

Analysis object	Perspective	Thinking	Relationship	Scenario	Mode
Related research	Manufacturing system optimization in the context of the “internet+” (Zhang and Li, 2017)	Systems thinking in manufacturing (Eijnatten et al., 2007) Collaborative systems thinking (Lamb and Rhodes, 2010)	Coordinated scheduling of human resources, product collaborative design and information resources (Antti et al., 2020)	Exploration of synergy strategies, influencing factors, off-site processes, improved organizational synergy capabilities and development paths (Mincuzzi et al., 2019)	Ways of building shared platforms, cloud manufacturing, intelligent manufacturing and collaborative interaction models (Guo et al., 2000)
Our research	Manufacturing system optimization in the context of “internet+”	Systematic collaborative manufacturing thinking	Complete internal and macro manufacturing enterprise processes	Quantitative analysis of the interaction between intrafirm activities and macro manufacturing firms	Combination of the causal relationship and flow diagrams of system dynamics to quantitatively and jointly analyze the influencing relationship along the entire system

Based on the systematic research perspective of the manufacturing industry in the context of “Internet+”, systematic collaborative manufacturing thinking is used for research. First, this article deeply discusses the collaborative relationship in resource sharing. Based on the shared organizational system of a multiagent architecture, this article details the internal and manufacturing enterprise levels. Second, regarding the shared process, the current research focuses on the improvement of macro synergy efficiency. This article comprehensively explores the synergy of various enterprises and resources with the help of micro-enterprise individuals and macro-manufacturing scenarios to efficiently use resources and operate enterprise production activities. Third, so that enterprises can share resources on the platform, a certain quantitative analysis is needed to measure the impact of resource sharing on the enterprise and the entire manufacturing industry (a quantitative analysis refers to the impact of various factors in the manufacturing enterprise system). A specific quantitative analysis first uses system dynamics to establish the influencing relationship of the entire system. Furthermore, it uses a system causal diagram and flow graph quantification to study the influence of resource sharing on the efficiency of network collaborative manufacturing. This systematic quantitative analysis studies the interrelationships in detail and describes the influences between various factors numerically, which reduces the uncertainty of the heterogeneous resources and information asymmetry of each participant, accurately evaluates the influencing factors of collaborative manufacturing efficiency and is conducive to the rapid allocation of resources. Effective measures promote the efficiency of network collaborative manufacturing, improve the operating effectiveness of the entire manufacturing market, and make policy suggestions from the government’s macro-control level for the industry. Thus, such measures provide a basis for government departments to formulate control strategies.

Combining Table 7 and the above comparison and analysis yields the following conclusions.

System dynamics has certain advantages in measuring the efficiency of collaborative manufacturing. First, the modeling and simulation of system dynamics can more accurately reflect the causal relationship in the process of collaborative manufacturing efficiency. Most of the current research on collaborative manufacturing efficiency adopts cross-sectional research, which makes it difficult to reflect the causal relationship in the continuous change process of individuals (Vancouver et al., 2010). Second, placing the interacting micro-enterprise individuals and macro manufacturing under the same framework can more comprehensively reflect the generation mechanism of collaborative manufacturing efficiency. System dynamics can include the two in the same frame system and examine their role in the impact mechanism in the dynamic impact. Third, other research methods have difficulty simulating long-term evolution, and system dynamics can make up for this defect. Using system dynamics to solve problems is beneficial to accurately quantify and reflect the complex connections in the entire system and further explore how to maximize the overall degree of synergy.

### Limitations and Challenges

Shared platforms have been widely used to solve the problem of resource sharing, but an important emerging challenge is measuring the degree of collaboration between enterprises in the process of resource sharing. The research on collaborative manufacturing mainly focuses on the manufacturing enterprise relationships among resource sharing, production, and operation. In addition, it also focuses on measuring and comparing their impact among them. However, there is a lack of quantitative analyses and exploration of the degree of synergy in the research results.

Systems thinking is an important scientific mode. In recent years, system dynamics research has focused on sustainable development, policy simulation, urban rail transit, the supply chain, and network public opinion. Few related studies have examined manufacturing progress. At present, the related research on collaborative manufacturing lacks the research-systems thinking perspective.

### Recommendations

The results suggest the following recommendations.

First, compared with other industries, the manufacturing industry, especially the large-scale manufacturing industry, works in a relatively backward combination. The reasons are various. In this article, the cloud platform constructed by the multiagent system provides the direction for manufacturing + Internet. More importantly, manufacturing enterprises should increase their trust and openness and provide practical experience for directional exploration.

Second, manufacturing enterprises must expand their communication circle, share resources, and increase trading opportunities. Second, enterprises need to constantly devote themselves to innovation and development to further improve production efficiency through technological reform. In the increasingly competitive enterprise environment, more profits can be guaranteed.

Third, from a macro perspective, the impact is not intuitive but is very important for the government and industry macro-control of economic efficiency and collaborative manufacturing efficiency. The quantity and quality of enterprises in the market must be adjusted, and investments must be made in scientific and technological innovation.

## Conclusion

This article focuses on systems thinking, breaks the boundaries between micro-enterprise individuals and macro manufacturing, and puts them under the same framework. Because of the difficulty in simulating long-term evolution with other research methods, we introduced a system dynamics simulation of the long-term efficiency of collaborative manufacturing. The specific changes can more comprehensively and systematically reflect the mechanism of collaborative manufacturing efficiency. Based on systematic collaborative manufacturing, by combining the macro-manufacturing industry with the micro-individual enterprises, this article sets the resource-sharing interaction scenario by building a cloud service platform to analyze the resource-sharing behavior among manufacturing enterprises. It also quantitatively studies the influencing factors of collaborative manufacturing efficiency with dynamic modeling and simulation analysis to propose improvement strategies, which will help make policy to improve creative performance and optimize the allocation of manufacturing resources.

Based on a multiagent shared platform, this article presents and quantifies the impact of resource sharing on the efficiency of network collaborative manufacturing through system dynamics and yields accurate influencing effects. However, this research focuses on only three systems in the macro-manufacturing industry. In actuality, the manufacturing industry and the relationships among its enterprises are more complex than these three systems alone indicate. Therefore, future research should continue to measure qualitative factors and explore relational systems and more accurate quantitative methods.

## Data Availability Statement

The original contributions presented in the study are included in the article/supplementary material, further inquiries can be directed to the corresponding author/s.

## Author Contributions

XXZ and XG wrote the section of the manuscript. SL and XHZ contributed to data curation and visualization. XXZ and HL contributed to manuscript revision. All authors approved the submitted version.

## Conflict of Interest

The authors declare that the research was conducted in the absence of any commercial or financial relationships that could be construed as a potential conflict of interest.

## Publisher’s Note

All claims expressed in this article are solely those of the authors and do not necessarily represent those of their affiliated organizations, or those of the publisher, the editors and the reviewers. Any product that may be evaluated in this article, or claim that may be made by its manufacturer, is not guaranteed or endorsed by the publisher.
